# Psoriasis

**Published:** 1896-11-21

**Authors:** George Parker

**Affiliations:** Assistant Physician Bristol General Hospital.


### PSORIASIS.

By George Parker, M.B., M.D.Cantab, Assistant Physician Bristol General Hospital.

It is remarkable that this disease, while one of the most common, is also one of the most incurable of all skin affections. Temporarily cured it often is, and sometimes the results last for years, but rarely is immunity lifelong. So rare are absolute cures that some authorities deny their existence. Exacerbations, particularly in spring and autumn, followed by more or less of disappearance of the symptoms, even in cases which are subject to no treatment, tend to render uncertain both the effect of remedies and the real termination of the disease. It may commence insidiously with one or two little hyperæmic patches on which scales develop. These may remain unaltered for years, or may at once multiply over a large area of the trunk and limbs. Perhaps no part of the skin is always and in every case exempt from its ravages, nor is it clear that any climate or race of men is entirely free from it. In Europe it is probably the most frequent of skin diseases, with the exception of eczema and acne. The extensor surfaces, or those most exposed to friction and pressure, suffer most—if we except the palms and soles, which are not commonly affected, and perhaps the covering of the ischial tuberosities. The mark of a waistband or scratches from the nails have been noticed by Nielsen (Sydenham Soc. Monographs, 1893) as frequent places for the eruption. Köbner, too, has made the singular observation that during a period of exacerbation a rough stroke with the nail on the skin is followed in eight or nine days by an eruption in the line of friction. Thus, while we know nothing of the cause of the disease, except its apparently strong hereditary character, exciting causes are frequently recorded. Thus, the first spots have occurred on the site of vaccination, of erysipelas, small-pox, tattooing, cupping, the pressure of a truss, blisters, burns, and other irritants. Yet Nielsen, whose collection of these facts is most interesting, remarks that the same sequence after irritation is true of many other skin affections. Opinion in this country is against any causal relation to other diseases. Neither the diathesis dartreuse, nerve disease, or syphilis have any proved share in its production. Moreover, it has no direct complications, nor does it affect the general health or functions, save that in chronic rheumatic patients the disease at times assumes a severe type, and that in any patients the cracked skin may be attacked by eczema or erysipelas. Kaposi remarks that in severe outbreaks indeed fever, gastric symptoms, and other troubles may for a while appear, but even these cases are rare.

As to the pathology, Jamieson considers the primary change to be a proliferation of the rete mucosum, while Kaposi holds that the first change is an inflammatory alteration commencing in the papillary layer, with dilatation of the vessels and cellular infiltration. Indeed, the earliest manifestation before any scales are seen, is a minute red spot, and every growing patch shows a hyperæmic border outside the scaling area.

An attack may vanish to a greater or less extent during the presence of a specific fever, during pregnancy, or in a chronic wasting disease, such as cancer or phthisis; but there are many exceptions, and after the fever is gone the psoriasis returns, even if it had temporarily disappeared. It was to be expected that a parasitic origin would be claimed in this as in so many other cases. Lang discovered a supposed microbe nearly twenty years ago, which was soon after shown to be illusory. Only once has it been possible to convey the disease by inoculation, and the possibility of infection as opposed to heredity is generally denied. Still Nielsen has shown that there is strong reason for believing in a parasitic source, and that infection does really take place. Several instances of a husband or wife developing the disease after cohabitation with a partner who suffered from it have been reported of late. Tenna saw three children who, without any hereditary taint, took it when attended by an infected nurse. Cantrell (*Med. Record*, May 2nd) speaks of a boy who was first attacked, while his mother and sister developed it later on, and also of a man who suffered for some years, and finally brought his mother for treatment when she became attacked for the first time. It may be answered that in such a common disease such extraordinary coincidences must occur, and that if these people had never lived together they would have developed the disease just the same. Thousands of children who have one parent affected show symptoms of the disease, while it is a rare exception for the other parent to develop it by an apparent contagion. Nielsen adduces as other proofs the apparently definite incubation period which takes place in Köbner's experiment; the form, growth, and mode of extension of the efflorescence; the fact that the most successful remedies are antiparasitic; and he urges that the rarity of complete cases depends on our want of thoroughness in removing every trace of the disease, and in disinfecting the entire skin as well as clothing and the comb and brushes. Indeed, the presence of the disease in other members of a family would on this theory be quite sufficient to prevent a cure. Among methods of treatment we find that internal remedies are comparatively useless. Arsenic, however, sometimes seems to give good results, while Haslund and his school have treated many patients with enormous doses of iodide of potash, obtaining a fairly rapid disappearance of the rash. Reliance is more often placed on external treatment, and it is noticeable that the most successful remedies have been, as we have said, parasiticides. Another matter of importance, as Kaposi remarks, is that all applications should be preceded by some process for the maceration and removal of the scales present on the skin and those newly forming. Baths, wet packs followed by friction, the rubbing in of oil, fat, vaseline, or soft soap for several days, or the application of fats on a dressing, or even caustic potash for very hard surfaces, will be found useful for removing the scales faster than they are produced. Then comes the time for the active remedies. The most efficacious is chrysalrobin, but it has the disadvantage of staining the linen and everything it touches. An ointment of 10 or even 20 per cent. strength is painted on the rash once a day, and bathing avoided as much as possible. In three or four days the spots begin to look paler and to disappear. It does not cause pain even on raw surfaces, but in a few cases causes boils or dermatitis. Jamieson recommends an ointment containing chrysalrobin 25 grains, salicylic acid 10 grains, ichthyol 25 grains, and green soap a drachm to the ounce. A

more cleanly method consists in coating the skin with gelatin or collodion containing 5 or 10 per cent. of chrysalrobin. When dry it may be painted over again with pure gelatin or collodion to prevent damage to the linen if possible. Pyrogallic acid may be substituted in Jamieson's ointment, and used for the head and face, as it does not discolou the skin so much. Beta naphthol ointment (10 per cent.) does not discolour the skin, and is perhaps the best for the face, but either of these substances, when applied over large surfaces, may be absorbed and cause serious nephritis. Kaposi, indeed, has applied naphthol ointment to the whole body several time a day for weeks, without ill effects, in hundreds of cases, but even he insists on the need of careful watching, and eschews alcoholic solutions. Aristol (one part in ten) may be used as an ointment or in collodion, or Hutchinson's ointment may be tried, containing 10 grains each of chrysalrob in liquor carbonis, and white precipitate to the ounce. Warm expectations were felt as to the thyroid treatment. Byrom Bramwell reported (July, 1894) thirteen cases improved out of eighteen. Phineas Abraham noticed that in a few cases it had a marked effect. Gordon Hill had similar results, but Malcolm Morris, Squire, and others found it useless, and in some instances serious constitutional effects followed. On the whole, it may be said to be worth trying in obstinate cases, and when any result is attained this may be supplemented by local remedies.

There are one or two types of psoriasis very difficult to deal with. Kaposi (*Med. Week*, May 8th) notices universal attacks in children, consisting of innumerable small foci over the whole skin. The application of almost any remedy to the entire body would cause intoxicaton, and he there gives arsenic with sulphur baths daily, a little simple ointment being rubbed in afterwards. In other cases there is so much inflammatory action that acute dermatitis occurs either at once or on the application of any remedy. Here he finds the cold wet pack of value, and in less severe cases the limbs may be treated by rubber bandages, Specific drugs must be stopped in any case until the inflammatory condition has passed away.

